# Association of Parental Stress and Early Childhood Caries

**Published:** 2009

**Authors:** Seyed Ebrahim Jabbarifar, Neda Ahmady, Seyed Ahmad Reza Sahafian, Fatemeh Samei, Shima Soheillipour

**Affiliations:** * Associate Professor, Pediatric Dentistry Department and Torabinejad Dental Research Center, School of Dentistry, Isfahan University of Medical Sciences, Isfahan, Iran; ** Assistant Professor, Pediatric Dentistry Department, School of Dentistry, Islamic Azad University, Isfahan, Iran; *** Dentist, Isfahan, Iran; **** Postgraduate Student of Clinical Psychology, Training & Psychology Science School, University of Isfahan, Isfahan, Iran; ***** PhD Student, Dental Public Health, King’s College, London, United Kingdom

**Keywords:** Dental caries, Dental stress analysis, Oral health

## Abstract

**Background:**

Little research has been carried out on whether the parental stress affects children’s oral health in general and dental caries in particular. This study aimed to investigate the association between parental stress and early childhood caries (ECC).

**Methods:**

A cross-sectional study was designed that included 250 children of 4-6 year-old; 127 ones attended the pediatric department of Isfahan School of Dentistry who had early childhood caries and a comparison group of 123 caries free children attended five kindergartens and pre-schools in Isfahan city. Clinical examinations were conducted to evaluate the caries status. The parents of the two study groups completed the self-administrated long form of the Parenting Stress Index questionnaire. Details of their socio-demographic status were gathered too. The collected data were analyzed by SPSS version 11.5. The nonparametric Mantel-Haenszel test for correlation statistics was used to determine bivariate associations between total parenting stress and their domains scores in the two groups; i.e., those with early childhood caries and the caries free group.

**Results:**

Mean score of PSI in the early childhood caries and caries free group were 286.66 ± 66.26 and 273.87 ± 31.03, respectively. There was not any significant relationship between total parental stress and ECC. The scores of the following domains of PSI demonstrated significant differences between ECC and CF groups: child reinforcement, child distractibility, child deficit attention, life stress and relationship with spouse (P = 0.01, 0.01, 0.001, 0.005 respectively).

**Conclusion:**

Findings of this study did not show any significant association between total parenting stress score and prevalence of early childhood caries.

## Introduction

Early childhood caries (ECC) is prevalent in 2-6 year-old preschool children.^1^–^3^ Severe early child-hood caries is more common among lower socioe-conomic groups and deprived ethnic minorities.^4^–^7^ Most studies of the determining causes of ECC used biological models.^8^–^11^ A few studies have explored the psychosocial determinants.^12^,^13^ In cross sectional studies with biopsychosocial models and incorporating parental low selfefficacy, stressful life events, stress level, knowledge, and beliefs have been reported to have a significant association with ECC.^12^–^14^

General stressors affect general and dental health. Stress and stressors have emerged in recent decades as variables of considerable interest in the examination of health and disease. Behaviours associated with general parental stress include inconsistency, increased negative communication, decreased monitoring and supervision, setting vague value rules, limits on behaviors, being more reactive and less proactive and engaging in increasing harsh disciplinary. Parenting stress, family environment stress, occupational and other aspects of stress are over lapped and interrelated.^15^,^16^

Therefore, it is surprising that psychosocial investigations have not extensively studied the relationship between parental stress and ECC. In view of the prevalent and varied effects of parenting stress, there is need for practitioners and researchers to explore the dynamic pattern and epidemiology of stress in the parenting role. Primary functions of parenting stress are initially identification screening, individual diagnostic assessment, prepost measures of interventional effectiveness, and measurements of studying effects of stress on parenting behaviors and other psychological factors and interactions.^17^–^19^

Stress in parents affects their behaviors and has negative effects on their children. Stressed parents are likely to respond negatively to the stress in their child. Within the context of the family stress, parenting stress has been divided into stress of the child, which refers to those qualities in the child that make it difficult and stressful for the parent to fulfill their parental roles in providing child’s needs. On the other hand, stress from parents and family characteristics refers to those emotional, financial, acute and chronic life stressors and events and potential dysfunctions that relates to different dimensions of parenting function. Effects of parenting stress are prevalent and varied. Higher parenting stress was associated with greater depressive symptoms, higher rates of use of medical services, illness, ignorance and neglectful behaviors as well as perception of own health status. At an interpersonal level, parenting stress has altered the marital relationship and resulted in decline of marital adjustment and training of children.^15^,^16^

The standardized Parenting Stress Index (PSI) is used in primary health care settings by clinicians, nurses and researchers as a valid and reliable measure of parenting stress. The PSI assesses stress related to the care givers perception of the child, selfperception of competence and mood and the competence of the parent-child dyad. In Iran, parenting can be a stressful task because of the factors such as financial problems, work status, overcrowding, safety, disasters, long-standing diseases, uncertainties and non-insurance. Untreated severe early childhood caries may affect the parents stress levels because the child may experience a dental abscess, pain, sleeplessness, reluctance to eat, retardation in development and growth and failure to thrive.^16^,^17^

The effect of caregiver’s and family stress on the child’s well-being and dental caries has been studied using the PSI. Studies on the relationship between dental caries in preschool children and parenting stress have reported inconsistent results.^18^

LaValle et al^19^ studied the effect of parental stress on oral health of their children. They found that the primary care-givers who were younger, less educated and more economically stressed reported a low child domain score on PSI. Also, a significant relationship between parenting stress and environmental risk factors has been reported.^20^

Quinonez et al^21^ and Tang et al^22^ found a significant association between parenting stress score and parent-child dysfunction interaction. There was a relationship between parent-child dysfunctional interaction and female caregiver education, child birth order, ethnicity, culture and belief.

The objective of this study was to investigate whether there is an association between parental stress and early childhood caries in 4-6 year-old children in Isfahanian families.

## Materials and Methods

The study protocol was approved by the Research Ethics Committee of Isfahan University of Medical Sciences. Formal consents of parents were obtained before commencing the study.

Two disparate populations were included in the study. One was a population of 127 parents and their 4-6 year-old children attending the Isfahan Pediatric Dentistry department for dental treatment. The other population included 123 children of 4-6 year-old recruited from five kindergartens close to the Isfahan School of Dentistry.

Children’s caries experience was recorded by a trained examiner using the WHO criteria, with a disposable mouth flat mirror and blunt explorer without drying the teeth, by ordinal room light. Infection control guidelines such as using disposable examination gloves and plastic sleeves were applied.

Parents of the two groups, generally the mother, concurrently completed the long form of PSI questionnaire.^3^ They were blinded to the clinical data of their children. The PSI contains 101 questions with 5 degree Likert type scoring system and 19 questions with yes/no response format. This index has fourteen domains in dyad of child-parents. Child characteristic domains are child acceptability, adaptability, demandingness, mood, distractibility and child reinforcement parent. Parent characteristic domains include depression, attachment, restriction parental role, competence, relation with spouse and health. PSI has a list of l9 life stressors such as accident, migration, jobless occasion, financial problem, marital status, death of closed relatives, economical break–downs, bereavement, divorce and happiness occasions.^3^

The collected data were analyzed by SPSS version 11.5. The descriptive statistics were recorded. The nonparametric Mantel-Haenszel test for correlation statistics was used to determine bivariate associations between total parenting stress and their domains scores in the two groups; i.e., those with early childhood caries (ECC) and the caries free group.

## Results

Two hundred and fifty 4-6 year-old children and their parents were enrolled in the study. The dmft in the ECC group was 2.6 ±3.15and there was no caries in the caries free group. Table 1 shows the distribution of socio-demographic characteristics of parents in ECC and caries free groups and the differences between them. There were no significant differences between the two groups in occupation and educational levels of parents. A higher percentage of caries free group had received fluoride therapy compared with ECC group.

**Table 1:** Distribution of socio-demographic characteristics of parents in the two populations.

		ECC%	Caries free%	P-value
Parent’s Occupation	Private	41.8	41.6	
	Housewives	17.4	16.4	N.S
	Employed by government	40	39	
Parent education level	Primary	35.5	29.1	
	Secondary	54.3	44.7	N.S
	Tertiary	8.2	14.2	
Fluoride therapy	Yes	11.0	30.1	P = 0.03
	No	89.0	69.9	
Medication	Yes	12.0	13.0	N.S
	No	88.0	87.0	

The distribution of oral hygiene status (good, fair, improper) in ECC and caries free group were 11.8, 44.9, 43.3 and 64.5, 26.6, 5.9 percent, respectively.

There were no significant differences in scores of the following domains between ECC and caries free group’s: child mood, parent sense of competence, child adaptability, parent attachment, restriction imposed by parental role, parental social isolation, parent health, child demandingness and parent depression. The scores of the following domains demonstrated significant differences between ECC and caries free groups: child reinforcement, child distractibility, child deficit attention, life stress and relationship with spouse (Table 2). There was not any relationship between dental trauma, used medications, hospital admission, dental flossing and dental caries.

**Table 2:** Mean and SD scores of Parental Stress Index and its different domains in ECC and caries free groups.

PSI Domains	ECC Mean ± SD	Caries free Mean ± SD	P-value
Child Reinforcement	13.96 ± 2.28	16.11 ± 9.05	*P = 0.01
Child Mood	132.63 ± 2.83	12.06 ± 3.57	P = 0.06
Child Distractibility	18.4±4.84	17.3 ± 3.97	*P = 0.01
Parental Sense of Competence	34.38 ± 6.27	33.90 ± 5.45	P = 0.52
Child Adaptability	31.20 ± 5.16	31.87 ± 4.45	P = 0.27
Parent Attachment	17.53 ± 3.43	17.33 ± 6.43	P = 0.75
Restriction Imposed by Parental Role	19.81 ± 5.47	19.83 ± 4.73	P = 0.97
Parental Social Isolation	14.66 ± 3.62	14.39 ± 3.78	P = 0.56
Parent Health	14.05 ± 3.65	13.60 ± 3.17	P = 0.30
Child Demandingness	23.15 ± 5.06	23.87 ± 5.71	P = 0.29
Child Deficit Attachment	26.33 ± 6.81	25.08 ± 5.35	P = 0.05
Life Stress	8.63 ± 6.81	5.91 ± 5.7	*P = 0.001
Parent Depression	25.25 ± 6.70	24.80 ± 5.26	P = 0.56
Relationship with Spouse	19.72 ± 4.33	18.02 ±5.11	*P = 0.005
Total Parental Stress Index (PSI)	286.66 ± 66.29	273.87 ±31.03	P = 0.05

* Significant

## Discussion

Total parenting stress was not significantly different between ECC and caries free group. The findings from the present study did not show any relationship between caries and higher parenting stress. But, there was direct relationship between general life stressors and ECC.

Also in our sample, relation with spouse, reinforcement, acceptability and total parenting stress scores between the two groups were significantly different.

Frank et a1^13^ found that fathers in poor families have more stress and showed a strong relationship between stress and child health status.

Moreover, a psycho-social family status of parents affects attachment levels of parental stress and children.

Finlayson et al^17^ and Hallet et al^18^ demonstrated a relationship between parent child dysfunction, interaction, female caregiver, education level, number of children and ethnicities.

Lavalla et al^19^ reported association between some aspects of parenting stress and oral health. Sanders et al^20^ in a study in Australia found significant relationship between parenting stress and injury prevention, child development, health services use and environmental risk factors.

A parent who does not expect much from a child is more likely to view the child easy to manage. Parents who place high demands on their children, always instructing their children to clean their rooms and doing their home works, brushing their teeth and brushing their hair, may see even minor deviations from these expectations as difficult and demandingness child. Children who do not display behaviors described as distractible are children who are considered easy to care. Socioeconomic status was not a significant variable in the two groups.

This finding may be due to the fact that most of the parents in our study were living in the same residential area (close to Isfahan Dental School). Examination of care givers with a broader range of family residential areas would provide a stronger test impact of socio-economic on child dental health and parental stress. Although our findings were from a cross-sectional study and need to be confirmed by longitudinal studies, the present study was the first one that investigated the association between parental stress and dental caries in Iranian Families.

Quinonez^21^ and Tang et al^22^ used the short form of parenting stress index to examine the association between parenting stress and development of early childhood caries in Australia. They observed a significant association between parenting stress and ECC. This finding is not compatible with our results.

The importance of parenting stress as a risk factor or mediator in the natural history of children diseases like early childhood caries has been reported by pediatricians and oral health researchers.^22^–^24^

In Kristinsen’s^25^ study, negative association between social desirability and extent of dental caries has been reported.

In our study, parents with high level of stress and with high dental caries of the children expressed their children’s dental health status as proper. We think parents are biased in reporting oral health status of their children.

More et a1^26^ showed that 30% of parents in stressful family environments exhibit low levels of engagement in home dental care, high levels of ill health behavior and emotional problems, and three times more parent aggression. Furthermore, one of every five children lives in a stressful family environment with two or more stressors such as inability to pay bills or obtain proper foods or having no health insurance.

Alemagno et al^27^ demonstrated that parents under stress may need social support and ways to develop a working support network to help prevent injury to their young children.

In our study, we did not measure the details of financial status of families but there was no significant difference between the educational levels of parents in caries free and ECC groups.

Parents and families with high stress levels did not support their children. Psychosocial family environment causes psychological reactions and provokes physiologic and pathologic events. Higher total parenting stress was significantly associated with specific demographic variables including lower female caregiver education, single parent and higher birth order of children.

Many parents in the studies of Amin et al^28^ and Leung et al^29^ reported that the stress of daily life was a barrier to properly pay attention to the health care of their children’s teeth. Types of stress in the later study were economical, sociodemographic, number of children and marital status. Also, a few oral health studies have explored the potential association among parenting stress, general health, knowledge, attitude, children’s oral health and diseases. In our study, these issues were not examined.

Other limitations in this study included the small convenient sample size and inherent limitations of caries status (caries, no caries) used to measure early childhood caries. It is necessary to design life course cohorts to evaluate cause and affect relationship between ECC and parenting (fatherhood, motherhood and family) stress. Lack of standardized diagnostic criteria for measurement and report of ECC in literature makes it difficult to compare results of different studies.

These shortcomings introduce potential sources of limits of generalizability of our findings. Different findings in this study compared with other studies are attributed to different samples, contexts, and methodology

## Conclusion

Findings of this study did not show any significant association between total parenting stress score and prevalence of early childhood caries. However, some domains of parenting stress may increase the possibility of dental caries in their children.

Our findings suggested the need to conduct longitudinal prospective and life course studies to give proper consideration to temporal aspects of early childhood caries and to clarify the results obtained on the relationship and strengths of parenting stress and oral health status in children.

## References

[1] McDonald RE, Avery DR, Dean JE (2004). Dentistry for the child and adolescent.

[2] Abidin RR (1983). Parenting stress and the utilization of pediatric services. Child Health Care.

[3] Stora JB (1993). Le stress.

[4] Jabbarifar S E, Sadeghian S (1999). Prevalence of nursing caries and related factors. Journal of Research in Medical Sciences, Isfahan.

[5] Acs G, Ng MW (2002). Early childhood caries and well being. Pediatr Dent.

[6] Jabbarifar S E, Abedi M R (2001). The association emotional Interactions among mother and child with prevalence nursing caries. Journal of Research in Medical Sciences, Isfahan.

[7] Litt MD, Reisine S, Tinanoff N (1995). Multidimensional causal model of dental caries development in lowincome preschool children. Public Health Rep.

[8] Ayhan H, Suskan E, Yildirim S (1996). The effect of nursing or rampant caries on height, body weight and head circumference. J Clin Pediatr Dent.

[9] Sheiham A (2006). Dental caries affects body weight, growth and quality of life in pre-school children. Br Dent J.

[10] Law CS (2007). The impact of changing parenting styles on the advancement of pediatric oral health. J Calif Dent Assoc.

[11] Jabbarifar S E, Makarem A (2006). Introduction to behaviour sciences and oral health.

[12] De Soet JJ, Van Gemert-Schriks MCM, Laine ML, Van Amerongen WE, Morré SA, Van Winkelhoff AJ (2008). Host and microbiological factors related to dental caries development. Caries Res.

[13] Frank SJ, Olmsted CL, Wagner AE, Laub CC, Freeark K, Breitzer GM (1991). Child illness, the parenting alliance, and parenting stress. J Pediatr Psychol.

[14] Fisher Owens SA, Gansky SA, Platt LJ, Weintraub JA, Soobader MJ, Bramlett MD (2007). Influences on children’s oral health: a conceptual model. Pediatrics.

[15] Tinanoff N, O’Sullivan DM (1997). Early childhood caries: overview and recent findings. Pediatr Dent.

[16] Harrison R (2003). Oral health promotion for high-risk children: case studies from British Columbia. J Can Dent Assoc.

[17] Finlayson TL, Siefert K, Ismail AI, Sohn W (2007). Psychosocial factors and early childhood caries among low-income African-American children in Detroit. Community Dent Oral Epidemiol.

[18] Hallett KB, O’Rourke PK (2003). Social and behavioural determinants of early childhood caries. Aust Dent J.

[19] LaValle PS, Glaros A, Bohaty B, McCunniff M (2000). The effect of parental stress on the oral health of children. Journal of Clinical Psychology in Medical Set-tings.

[20] Sanders AE, Spencer AJ (2005). Childhood circumstances, psychosocial factors and the social impact of adult oral health. Community Dent Oral Epidemiol.

[21] Quinonez RB, Keels MA, Vann WF, McIver FT, Heller K, Whitt JK (2001). Early childhood caries: analysis of psychosocial and biological factors in a high-risk population. Caries Res.

[22] Tang C, Quinonez RB, Hallett K, Lee JY, Whitt JK (2005). Examining the association between parenting stress and the development of early childhood caries. Community Dent Oral Epidemiol.

[23] Tam KK, Chan YC, Max Wong CK (2006). Validation of the parenting stress index among Chinese mothers in Hong Kong. Journal of Community Psychology.

[24] Mash EJ, Johnston C, Kovitz K (1983). A comparison of the mother child interactions of physically abused and non-abused children during play and task situa-tions. Journal of Clinical Child & Adolescent Psychology.

[25] Kristiansen CM, Harding CM (1984). The social desirability of preventive health behavior. Public Health Rep.

[26] More K A, Vandivere S (2000). Stressful family lives: child and parent well-being. New federalism: national survey of America’s families, series B, No. B-17; assessing the New Federalism. Urban Institute.

[27] Alemagno SA, Niles SA, Shaffer-King P, Miller WJ (2008). Promoting health and preventing injury in preschool children: the role of parenting stress. Early child-hood research and practice.

[28] Amin MS, Harrison RL, Weinstein P (2006). A qualitative look at parents’ experience of their child’s dental general anaesthesia. International Journal of Paediatric Dentistry.

[29] Leung SSL, Leung C, Chan R (2007). Perceived child behavior problems, Parenting stress, and marital satisfaction: Comparison of new arrival and local parents of preschool children in Hong Kong. Med J.

